# Embedding 21st century employability into assessment and feedback practice through a student–staff partnership

**DOI:** 10.1099/acmi.0.000329

**Published:** 2022-03-04

**Authors:** Dzachary Zainudden, Miranda Broom, Anna Nousek-McGregor, Fiona Stubbs, Nicola Veitch

**Affiliations:** ^1^​ School of Life Sciences, MVLS, University of Glasgow, Glasgow, UK

**Keywords:** embedding employability, microbiology, immunology, digital teaching, assessment and feedback

## Abstract

In-course assessments are an essential part of any coursework because they represent both a physical output of the skills and knowledge acquired at university, but also have the role of supporting student transitions into prospective careers. Therefore, assessments could be used as a conduit to encourage student awareness of the skills needed for the workplace and verify their attainment of these skills, particularly at foundation levels within a degree. Within a second-year Microbiology and Immunology course, students struggled to engage with standalone timetabled careers-related sessions, yet they showed enthusiasm when employability was embedded into assessments. A staff–student partnership project explored these issues, with the overall aim of understanding how to effectively embed employability skills into assessment and feedback and support students positioning themselves for the future. Through a focus group, this project investigated the reasons for low student engagement with timetabled employability sessions and used student views to develop digital initiatives and 21st-century competencies that could be applied more widely within assessment and feedback practice. These initiatives were then implemented as pre-session self-directed activities, with the objective of helping students to link course feedback with employment skills and future career planning, followed by a newly developed in-class reflective feedback session that allowed students time to consider what skills they have developed and make links with future careers. Project evaluation was conducted using a quantitative survey of the students involved.

## Introduction

Currently, there is a gap between the skills of university graduates and those applied in the workplace [1–3]. Data from Oxford Economics reveal that, over the forecast period (2017–2027 inclusive), future employment in Scotland within the Life Sciences sector is expected to grow by 5 % (equivalent to 1100 new jobs) and by 2027, 63 % of these will be higher-level roles [4]. Discrepancies in debates on whether the responsibility of instilling employability skills lies in the university or the employer can impede the quality of graduates after completing their academic careers. According to Cranmer [5], it was argued that universities are not liable to ‘provide training’, and guaranteeing employment prospects would be dependent on the ‘academic quality’ of graduates in conjunction with their degree of choice.

In contrast, Mason *et al.* [6] argued that graduates would benefit from gaining skills at the beginning of employment. Newly employed individuals are more likely to attain and practise a specific set of skills tailored for their occupation during orientation at the workplace rather than in the classroom where they are equipped with transferable yet non-specialized skills.

**Fig. 1.**
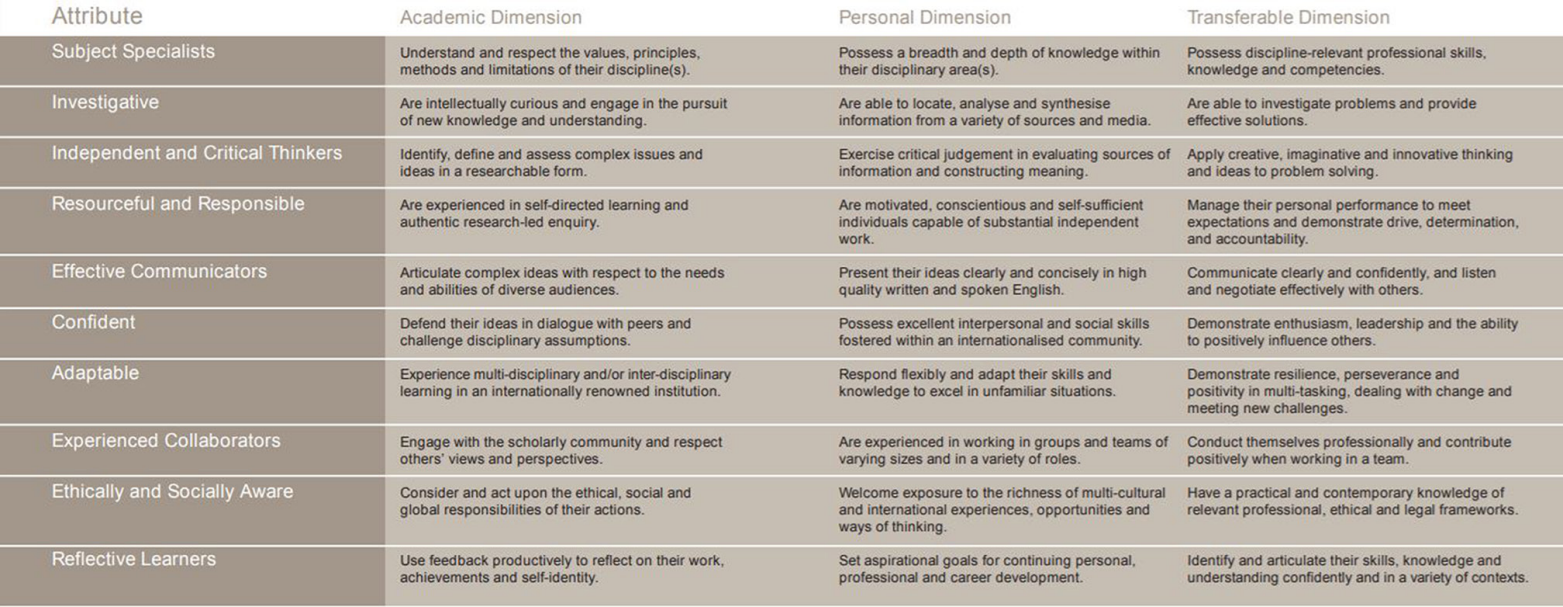
Glasgow Graduate Attributes Matrix.

Therefore, universities have implemented several strategies to support employability in their graduates [7]. The University of Glasgow (UofG) developed a Glasgow Graduate Attributes matrix that defined academic abilities, personal qualities and transferable skills students would develop while attending the University (Fig. 1). A report on digital literacy recommends that employability should inform the development of effective assessment methods, and the development of digital resources is a perfect opportunity to enhance ‘built-in employability’ [8, 9] within undergraduate degree programmes.

Current trends suggest that assessment strategies should be revised to align with transferable skills for the workplace, as demonstrated by the university-wide interest in revising assessment practice [3]. Similarly, because students receive detailed feedback on their assessments, using artefacts developed for assessment will mean that students have been supported with the evidence in their career search.

This project aimed to enhance assessment practices in a Year 2 Microbiology and Immunology course in the School of Life Sciences (SoLS) at the UofG to strengthen student employability skills. This course normally has approximately 200 students. The approaches were designed in partnership with former students from the course, and therefore this project possessed the unique ability to deliver a genuine student voice both on employability concerns and on the role of assessment.

## Project development

**Fig. 2.**
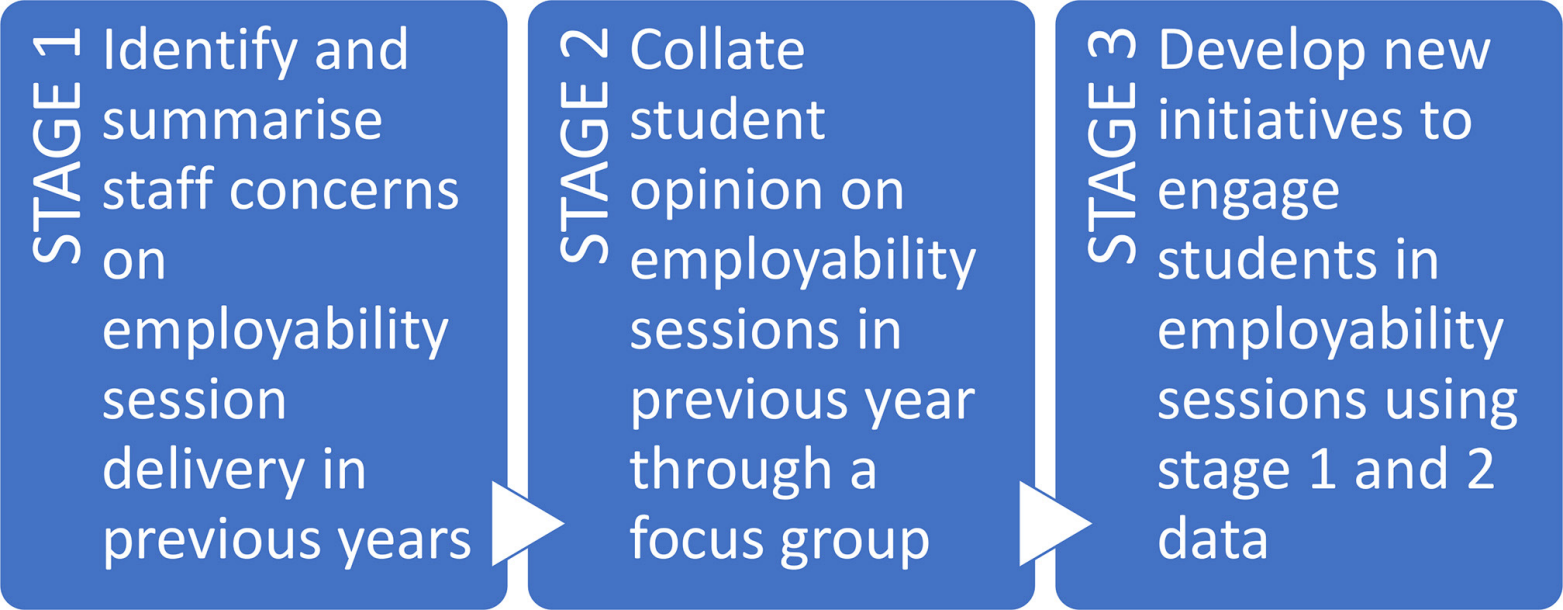
Flow chart of the project development plan.

A staff–student partnership project was funded through a Learning and Teaching Development Grant to engage with key stakeholders around careers-related sessions, with the overall aim of understanding how to effectively embed employability skills into assessment and feedback, supporting student development (Fig. 2). Two Year 3 Microbiology BSc (Hons) students were recruited as student interns for 1 year to collaborate with the Microbiology and Immunology course coordinator, the SoLS Employability lead and the SoLS Careers Manager.

**Fig. 3.**
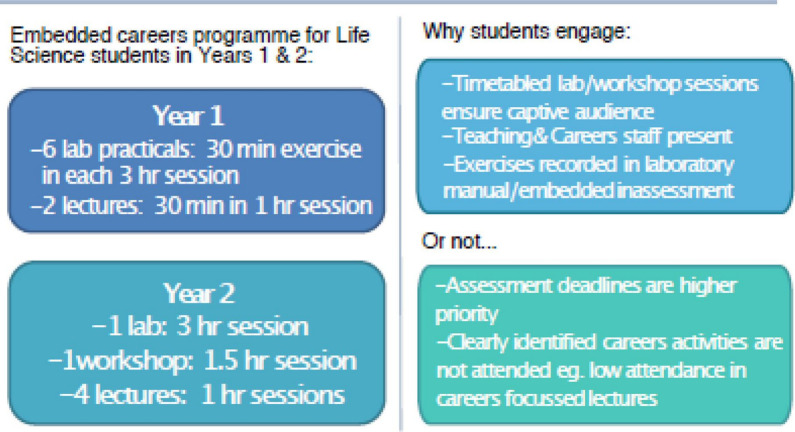
Staff-focused discussion outcomes, summarizing level 1 and 2 employability-based sessions and perceived barriers to engagement.

Initially, the two students worked with the SoLS careers manager to identify what employability-based sessions were delivered in Years 1 and 2 of Life Science degree programmes within the UofG. Discussions with course coordinators and the careers manager led to suggestions as to why students did and did not engage with these sessions (Fig. 3). One main outcome of this research was that students struggled to engage with stand-alone employability-based sessions, yet they showed enthusiasm when employability was embedded into assessments.

Following this initial phase of research, the two student interns ran a focus group to investigate reasons for low student engagement with timetabled employability sessions. Eight Year 3 students who had completed the Year 2 course the previous year volunteered to be part of the focus group and were asked questions around this topic, and their responses were recorded and transcribed. These students were selected as they had completed the Microbiology and Immunology course the previous year so could feedback on their experiences of employability sessions. Thematic analysis was used to identify the key themes shown in Table 1. Students found shorter employability sessions integrated into the curriculum to be a more effective method to deliver careers-based information. They noted their desire for guest speakers from industries related to their course subject to learn about external opportunities. This allows students to gain insight into career options and consequently instil themselves with motivation for future employability. Furthermore, students suggested guest speaker events require multi-platform promotion to target a wider audience and encourage engagement. Students expressed interest in assessments that demonstrate transferable skills beneficial to employability such as lab reports and group presentations. Lab reports promote writing, analysis and research skills directly related to research careers while group presentations stimulate communication, teamwork and problem-solving skills. In addition, students want feedback on assessments to be consistent, structured, meaningful and related to skills development.

**Table 1.** Focus group questions and outcomes of student discussion

Focus group question	Key themes arising from student discussion
What is the most effective way to deliver employability skills?	Short sessions integrated into practical lab sessions; invite guest speakers from industry from experts in the field
What motivates students to engage with employability sessions?	To learn about external opportunities
How can we use assessment and feedback to enhance employability skills development?	Feedback needs to be written in a style associated with skills development and feedforward into next assessment; improve student communication around feedback

## Outcomes

Students’ views collated from the focus group were used to develop a new digital initiative linked to a student-centred workshop, with an emphasis on building a portfolio around developing 21st-century competencies. This initiative was implemented as a self-directed, flipped learning activity helping students associate course feedback with employment skills and future career planning. Prior to the scheduled class, the level 2 Microbiology and Immunology students were given access to ‘Assessing for Employability’, a web-based tool designed in Articulate Storyline (version 2, New York) and accessed through the university’s Virtual Learning Environment to increase awareness of the diverse digital capabilities required in the 21st century job market and enable students to explore how skills development was embedded into the coursework, particularly through summative assessments. They were asked to reflect on their personal career interests and then evaluate skills developed during their Microbiology and Immunology course that align with that career direction (Supplementary data 1). At the end of the Assessing for Employability tool, students could submit feedback on the tool as well as their perspective on employability skills. They were given a week to review the tool, after which they had a 1 h session to complete an in-class reflective feedback session. Students were asked to bring their feedback to the session and work in groups to identify what skills they had developed. They then submitted answers via the Mentimeter electronic response system (version 1, Stockholm).

**Fig. 4.**
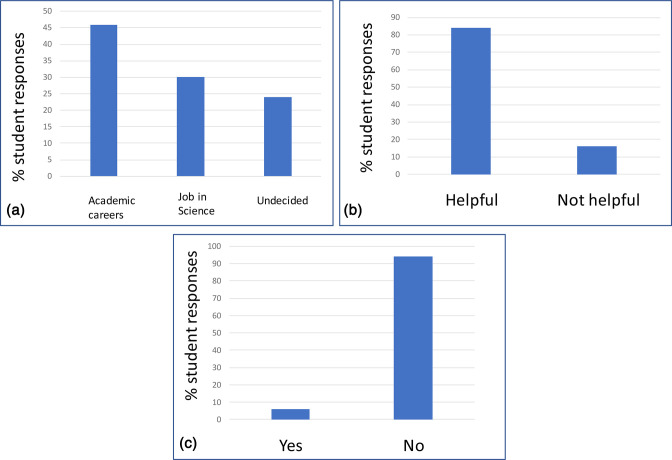
Student responses to the Assessing for Employability Tool survey. (a) Responses when asked what jobs students were interested in when graduating. (b) Responses when asked if they found the tool helpful for relating their degree work to post-university experience. (c) Responses when asked if students knew of the UofG Graduate Attributes.

Forty-four out of 200 Year 2 students taking the Microbiology and Immunology course attempted the web-based tool, with an average duration of 60 min between starting and stopping the resource. Of those, 13 completed the end-of-survey evaluation (Fig. 4). Approximately half of the students indicated an interest in academic careers (six students), while 30 % were interested in a job in science, and 24 % were undecided. When asked whether they found this resource helpful for relating their degree work to post-university experience, 84 % indicated a helpful category, with the remaining 16 % indicating it was unhelpful. The most concerning result from this evaluation was the lack of student understanding of graduate attributes; 92 % of participants responded they had either heard of them and did not know what they were or they knew what they are but had never referred to them in their academic career.

The second main outcome of this project was to effectively embed an external employer into the curriculum. The Scottish Environmental Protection Agency (SEPA) Microbiology leads were invited to give the students a lecture around the work they do, that linked to the laboratories the students took, examining local river water for bacterial contamination. SEPA reviewed the course’s lab practicals and suggested how to update experimental methodology with techniques used within their organization. Course lecturers and the career manager had a reciprocal visit to SEPA to discuss what work they were doing and to discuss work-related opportunities for students.

## Future suggestions

The Life Science job sector is an area of growth in Scotland with highly skilled workers required [4]. Based on conversations with recruiters, there are incidences of life science companies having difficulty recruiting the right candidates from graduates, suggesting that these non-technical skills are areas to focus on in class. These skills include general business and commercial skills, digital skills, together with communication and team working. This of course varies according to the role.

Central careers services continue to provide careers information, advice and guidance to students online and in-person via careers masterclasses, employer presentations, careers fairs, websites with resources, social media platforms and 1 : 1 career coaching. Employer presentations will be planned as both in-person and virtual careers events via online delivery, to provide students with flexible and inclusive options.

The future focus of careers input will be around developing connections and communities within disciplines that are directly informed by trends in the current labour market. Creating scalable, networking events for each of the Life Science subjects on LinkedIn is a key element for undergraduates developing connections with graduates and supportive communities. Students can start relationship building and creating strong career communities within and beyond campus. Timetabled sessions will be offered on creating a LinkedIn profile and using the features of the platform. Bespoke sessions in Life Science degree groups will be created so that current students can connect with alumni.

Within the Life Sciences sector, there continues to be significant demand for skilled workers across a range of occupations relating to COVID-19 testing, vaccine production and the wider supply chain, so introducing students to these employers is a focus for careers activities whether in class or from the central careers service. Future job opportunities for Life Science students are wide-ranging and include science and non-science roles. In general, the skills employers are looking for in all graduates vary but the top skills remain relatively consistent [10].

This study has shown that engaging Life Science students with employability elements as early in their degrees as Year 1 is important when considering careers delivery and requires it to be in a way that makes it easy for them to engage in careers education within their timetable, and preferably embedded into an academic session. It is hoped that they then see it as a critical part of the fabric of the student experience in all years rather than a resource that they seek when they approach graduation. Engaging students in this project allowed for inclusion of the student perspective, an initiative that has been extended by the recruitment of two Life Sciences interns with the School for the next academic year to engage students more fully in the Career Service’s agenda. Coupled with the creation of the SoLS Employability Group with 10 academic members of staff, this work has enabled the creation of a strong employability presence in the School, which will hopefully expand the potential to embed employability within the Life Sciences curricula and elsewhere within the University’s academic agenda.

## Supplementary Data

Supplementary material 1
