# Supplementary material for: Embedding 21st century employability into assessment and feedback practice through a student–staff partnership
Source: Access Microbiol. 2022 Mar 4;4(3):000329. doi: 10.1099/acmi.0.000329 (PMC9175971; doi:10.1099/acmi.0.000329)

Supplementary data 1: Assessing for Employability Tool, created in Articulate Storyline 2. Instructions for student self-assessment of skills for students when using the Assessing for Employability tool prior to their in-class feedback session.

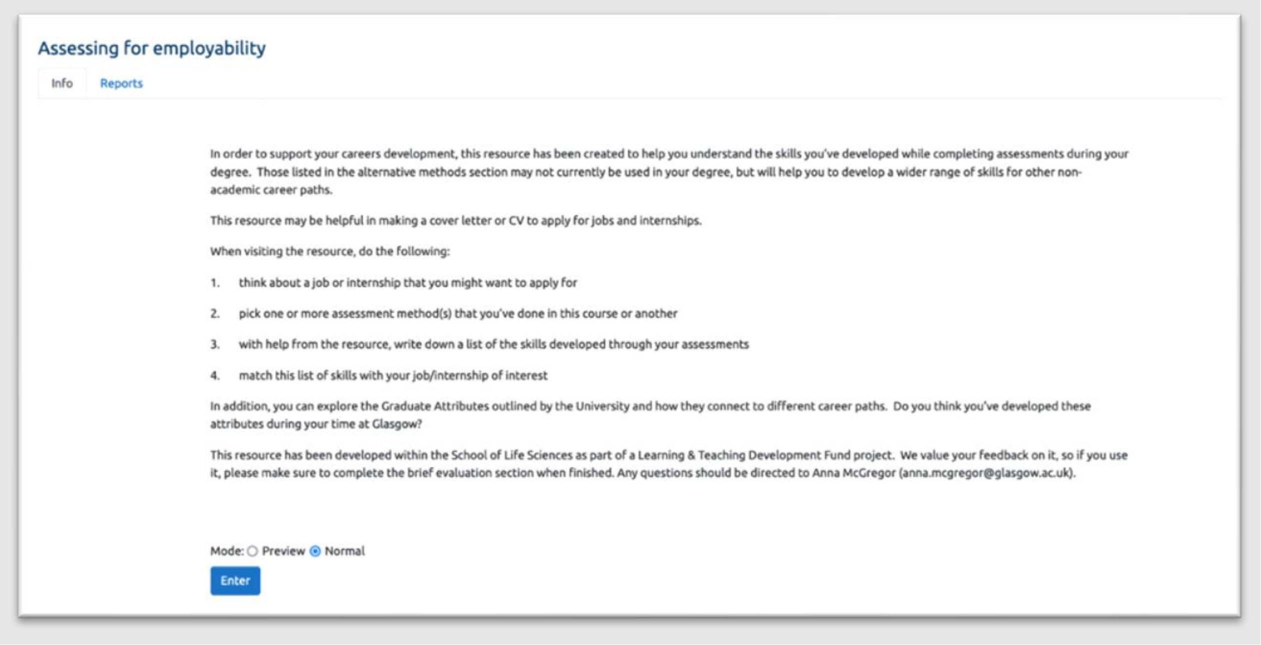

Supplement: Supplementary material 1 [file acmi-4-0329-s001.pdf]
